# Antidepressants overuse in primary care: prescription trends between 2010-2019 in Catalonia

**DOI:** 10.1192/j.eurpsy.2023.1151

**Published:** 2023-07-19

**Authors:** M. S. Gonzalez, G. anmella, E. vieta, M. Primé-Tous, X. Segú, A. Mas, D. Hidalgo

**Affiliations:** 1 IDIBAPS, CIBERSAM, Hospital Clínic; 2 IDIBAPS, CIBERSAM, Hospital Clínic Barcelona, Barcelona, Spain

## Abstract

**Introduction:**

Antidepressants (AD) are one of the most prescribed pharmacological treatments in developed countries. AD efficacy is well-proven in anxiety, depressive and other mental disorders, but their use is also common in individuals without psychiatric health conditions. Indeed, recent evidence reported an increase in AD prescription over the latest years. Concern has been raised on the overuse of AD in several countries, and societal policies and national guidelines have been developed to regulate their use in the general population.

Several factor might be used to explain this increase, including the more safety profile of new AD classes (i.e. SSRI, or vortioxetine) compared to old AD, a possible overall increase in the incidence of depressive and anxiety disorders, or their inappropriate prescription in mild conditions which could be managed without pharmacological treatment as first-step option in primary care (PC).

**Objectives:**

Explore AD prescription patterns in relation to mental health diagnoses and identify the most relevant factors involved in PC health systems. Understanding the variables influencing AD prescription would allow designing strategies and guidelines to make appropriate use of this pharmacological group in PC. As part of the PRESTO project (www.prestoclinic.cat), here we investigated the changes in frequency and the variables associated with AD prescription in a population-representative sample of people attending PC between 2010 –2019 in Catalonia, Spain.

**Methods:**

We retrieved AD prescription, sociodemographic, and health-related data using individual electronic health records from a population-representative sample (N=947.698) attending PC between 2010-2019. Prescription of AD was calculated using DHD (Defined Daily Doses per 1,000 inhabitants/day). We compared cumulative changes in DHD with cumulative changes in diagnoses with an indication for AD during the study period. We used Poisson regression to examine sociodemographic and health-related variables associated with AD prescription.

**Results:**

Both AD prescription and mental health diagnoses with an indication for AD gradually increased. At the end of the study period, DHD of AD prescriptions and mental health diagnoses with an indication for AD reached cumulative increases of 404% and 49% respectively. Female sex (incidence rate ratio (IRR)= 2.83), older age (IRR = 25.43), and lower socio-economic status (IRR= 1.35) were significantly associated with increased risk of being prescribed an AD.

**Conclusions:**

Our results from a large and representative cohort of patients confirm a steady increase of AD prescriptions that is not explained by a parallel increase in mental health diagnoses with an indication for AD. A trend on AD off-label and over-prescriptions in the PC system in Catalonia can be inferred from this dissociation.

**Disclosure of Interest:**

M. Gonzalez: None Declared, G. anmella Grant / Research support from: received CME-related honoraria, or consulting fees from Janssen-Cilag, Lundbeck, Lundbeck/Otsuka, and Angelini, with no financial or other relationship relevant to the subject of this article, E. vieta Grant / Research support from: received research support from or served as consultant, adviser or speaker for AB-Biotics, Abbott, Abbvie, Adamed, Angelini, Biogen, Celon, Dainippon Sumitomo Pharma, Ferrer, Gedeon Richter, GH Research, Glaxo SmithKline, Janssen, Lundbeck, Organon, Otsuka, Rovi, Sage pharmaceuticals, Sanofi-Aventis, Shire, Sunovion, Takeda, and Viatris, out of the submitted work, M. Primé-Tous: None Declared, X. Segú: None Declared, A. Mas: None Declared, D. Hidalgo Grant / Research support from: received CME-related honoraria or adviser from Abbott, Angelini, Janssen-Cilag and Ethypharm with no financial or other relationship relevant to the subject of this article.

